# Left Ventricular Systolic Dysfunction in Asymptomatic Marfan Syndrome Patients Is Related to the Severity of Gene Mutation: Insights from the Novel Three Dimensional Speckle Tracking Echocardiography

**DOI:** 10.1371/journal.pone.0124112

**Published:** 2015-04-22

**Authors:** Mohamed Abd El Rahman, Denise Haase, Axel Rentzsch, Julia Olchvary, Hans-Joachim Schäfers, Wolfram Henn, Stefan Wagenpfeil, Hashim Abdul-Khaliq

**Affiliations:** 1 Department of Pediatric Cardiology, Saarland University Hospital, Homburg/Saar, Germany; 2 Department of Pediatric Cardiology, Cairo University, Cairo, Egypt; 3 Department of Thoracic and Cardiovascular Surgery, Saarland University Hospital, Homburg/Saar, Germany; 4 Department of Human Genetics, Saarland University Hospital, Homburg/Saar, Germany; 5 Institute of Medical Biometry, Epidemiology and Medical Informatics, Saarland University Hospital, Homburg/Saar, Germany; University of Messina, ITALY

## Abstract

**Background:**

In asymptomatic Marfan syndrome (MFS) patients we evaluated the relationship between the types of fibrillin-1 (FBN1) gene mutation and possible altered left ventricular (LV) function as assessed by three-dimensional speckle tracking echocardiography (3D-STE).

**Methods and Results:**

Forty-five MFS patients (mean age 24±15 years) and 40 age-matched healthy controls were studied. Genetic evaluation for the FBN1 gene was carried on 32 MFS patients. Gene mutation (n = 15, 47%) was classified as mild when the mutation resulted in nearly normally functioning protein, while mutations resulting in abnormally function protein were considered to be severe (n = 17, 53%). All patients and controls underwent 3D-STE for evaluation of LV function by an echocardiographer blinded to the results of the genetic testing. Compared to controls, MFS patients had significantly lower 3D-STE derived LV ejection fraction (EF, 57.43±7.51 vs. 62.69±4.76%, p = 0.0001), global LV longitudinal strain (LS, 14.85±2.89 vs. 17.90±2.01%, p = 0.0001), global LV circumferential strain (CS, 13.93±2.81 vs. 16.82±2.17%, p = 0.0001) and global LV area strain (AS, 25.76±4.43 vs. 30.51±2.61%, p = 0.0001). Apart from the global LV LS all these parameters were significantly lower in patients with severe gene mutation than in those with mild mutation (p<0.05). In the multivariate linear regression analysis only the type of mutation had a significant influence on the 3D-STE derived LVEF (p = 0.017), global CS (p = 0.005) and global AS (p = 0.03).

**Conclusions:**

In asymptomatic MFS patients latent LV dysfunction can be detected using 3D STE. The LV dysfunction is mainly related to the severity of gene mutation, suggesting possible primary cardiomyopathy in MFS patients.

## Introduction

Marfan syndrome (MFS) is an autosomal-dominant genetic disorder of the connective tissue with an estimated prevalence of 1 in 5000 individuals worldwide [1, 2]. It is caused by mutations of the gene encoding the extracellular matrix (ECM) protein fibrillin-1 (FBN1) which is located on chromosome 15q21.3 [3]. The clinical diagnosis is based on the presence of major and minor criteria in different organ systems, as defined by the revised Ghent criteria [4]. Cardiovascular manifestations are common in MFS, most importantly in the form of aortic root dilatation, predisposing a patient to aortic dissection and rupture which constitute the main cause of mortality in this cohort [5]. Another common cardiovascular manifestation of MFS is mitral valve prolapse, which may cause severe mitral regurgitation requiring surgical intervention [6, 7]. Myocardial dysfunction in MFS patients was considered to be related primarily to aortic and mitral valve regurgitation and the surgical intervention was mainly on these valves and on the ascending aorta. However, there is still debate about whether a form of primary cardiomyopathy related to the FBN1 mutation exists among MFS patients [8, 9]. In addition, it is unclear whether there is an association between the severity of the mutation and the degree of ventricular dysfunction. This may be in part due to the lack of a non-invasive diagnostic tool that could evaluate possible latent regional as well as global ventricular dysfunction without any assumptions.

Three-dimensional (3D) echocardiography and 3D derived speckle tracking echocardiography (STE) are novel, reliable techniques for the evaluation of left ventricular (LV) left atrial (LA) performance [10–14]. These techniques are able to detect latent global and regional myocardial dysfunction, which cannot be detected by the conventional echocardiography methods.

The purposes of this study performed in a cohort of MFS patients were, first, to assess ventricular and atrial myocardial performance using these novel techniques and, second, to clarify the presence of a relation between LV dysfunction and the severity of gene mutation.

## Methods

### 1. Ethics statement

This study has been approved by the ethics committee in the federal state of Saarland, Germany (address: Faktoreistraße 4, DE66111 Saarbrücken, Germany); Statement date: August 11^th^, 2014, statement no. 131/14. Informed written consent was obtained from the patients or their parents.

### 2. Subject population

Forty-five consecutive Marfan patients were included in the study. They were included in the time interval between February 2012 and August 2013. All patients had a diagnosis based on the revised Ghent criteria [4]. They were selected on the basis that they were asymptomatic and were in sinus rhythm. The presence of significant mitral or aortic regurgitation was an exclusion criterion. Additional echocardiographic exclusion criteria were an echocardiographic window too poor to scan the ventricular or atrial walls adequately, grayscale image with a frame rate of less than 40 FPS and the presence of stationary reverberations or dropouts.

The age of the MFS patients was 25±15 years. Fourteen patients had been previously operated on because of severe aortic root dilatation. Their age at operation was 27±14 years. The period of follow-up was 3.5±3.7 years. MFS patients with previous surgery were statistically older than those without (20±13 vs. 35±16 years, p = 0,003).

Ultrasound data from the patient group were compared with those from 40 age-matched healthy controls. Their mean age was 20 ± 8 years.

The control group was made up of students in the medical school (most of them were friends of Dr Haase), daughters and sons of our medical staff and other volunteers.

### 3. Electrocardiogram (ECG)

All patients had a 12-lead surface ECG performed with a Siemens recorder (Siemens, Erlangen, Germany) at a speed of 50 mm/s and 1 mV/cm standardization. The QRS and the corrected QT intervals were measured in any lead.

### 4. Conventional echocardiographic examination

All patients were examined by echocardiography using a 2.5–3.5 MHz phased-array transducer with a Vingmed Vivid 9 ultrasound system (General Electrics, Fairfield, CT, USA). Initially, conventional echocardiography was done in accordance with the European Society of Echocardiography recommendations [15]. Tricuspid and mitral annular plane systolic excursion (TAPSE and MAPSE) were measured using two-dimensional echocardiographically guided M-mode recordings from the apical four-chamber view with the cursor placed at the free lateral wall of the tricuspid and mitral annulus. Significant mitral regurgitation was defined as a regurgitant jet of more than 4 cm ^2^ or more than 20% of the left atrial area. Mitral valve prolapse was defined as systolic displacement of one or both leaflets > 2mm from the mitral annulus plane in the LVOT view. Significant aortic regurgitation was defined as a ratio > 0.25 of the width of the regurgitant stream at the level of the aortic valve relative to the size of the left ventricular outflow tract measured in the parasternal long-axis view [9]. The aortic diameters were measured from leading edge to leading edge at the level of the aortic valve, aortic root and sinotubular junction in the parasternal long-axis view. When a sufficient echocardiographic window was available, the descending aorta diameter was measured in the subcostal long-axis view just before the origin of the celiac artery. Doppler assessment of LV diastolic function was analyzed as previously described [16]. The transmitral early (E-wave) and late (A-wave) diastolic velocities and diastolic time were recorded and the left ventricular Tei index was measured [17].

### 5. Assessment of ventricular and atrial function using real-time three-dimensional echocardiography (RT3DE)

RT3DE was performed from an apical four-chamber view using a 3D matrix array transducer (Vingmed Vivid 9 ultrasound system, General Electrics, Fairfield, CT, USA). A wide-angle acquisition “full volume” mode was used, in which 6 wedge-shaped sub-volumes were acquired for 6 consecutive cardiac cycles during a single breath-hold, resulting in a study in temporal resolution of 6 frames per cardiac cycle with a minimum frame rate of 42/s. Special care was taken to include the entire ventricular and atrial cavity in the 3D pyramidal volume.

Acquisitions were stored in a DICOM format and transferred to a separate workstation for offline data analysis. The images were further analyzed by an investigator who was blinded to the type of gene mutation. 3D wall motion tracking software 4D Auto LVQ (General Electrics Healthcare, Fairfield, CT, USA) and Tom Tec Imaging Systems (Unterschleissheim, Germany) were used to analyze the LV and LA respectively [13, 18].

The LV ejection fraction (LVEF) was calculated according to the formula LVEF = (LVEDV—LVESV)/ LVEDV. Global longitudinal, circumferential and radial strain was automatically calculated by the software [19]. In addition to these standard parameters the area strain, which integrates longitudinal and circumferential deformations, was calculated automatically [20].

For evaluation of the LA function, the LA endocardial border in the three planes was manually traced (pulmonary veins and coronary sinus and the appendages were excluded), starting the measurements in the frame with the largest atrial dimension, corresponding to ventricular end-systole, just before the opening of the atrio-ventricular valves. Then the frame with the smallest atrial dimension (at ventricular end-diastole, just before the closure of atrio-ventricular valves) is selected and the same manual tracing is applied. The software generates a time-volume curve. From the plotted LA time-volume the kick volume of the atria just before the P-wave was obtained. The reservoir function of the LA was calculated according to the formula ((LA volume max)–(LA volume kick)) / (LA volume max) and the pump function of the LA according to the formula ((LA volume kick)–(LA volume min)) / (LA volume kick), while the LA EF was calculated according to the formula LA EF = ((LA volume max)–(LA volume min)) / (LA volume max) [21, 13].

The recordings from 10 patients and 10 normal subjects were randomly selected and analyzed by two independent examiners to assess the inter-observer variability of the speckle tracking parameters related to ventricular deformation.

### 6. Statistical analysis

Statistical analyses were performed using SPSS 15.0 (SPSS Inc., Chicago, IL, USA). All continuous variables were tested for normality using the Kolmogorov-Smirnov test. The data are shown as mean ± SD or median and range if not normally distributed. Continuous variables with normal distribution were compared using the independent-samples T test. Mann-Whitney test was used to compare non-normally distributed continuous variables. Categorical variables with normal distribution were compared using the chi-square test. Correlations were evaluated using the Pearson correlation in normally distributed variables or Spearman correlation in non-normally distributed variables. Multivariate linear regression with forward and backward variable selection was used to determine the effect of age, aortic root diameter, surgical interference and severity of gene mutation on the LV ejection fraction derived from 3D echocardiography. A p value <0.05 was considered statistically significant. In the genetically tested patients (n = 32), the receiver-operating characteristic (ROC) curves were constructed for the prediction of severity of gene mutation. Inter-observer variability was calculated according to the formula (observer1–observer2) / [(observer1+observer2)/2] * 100%.

## Results

### 1. Demographic, clinical and genetic characteristics

Demographic and clinical characteristics are shown in Table 1. The MFS patients showed a slight female predominance and age of 24±15 (range 5–61) years. They were in sinus rhythm and had a significantly more rapid heart rate than controls (p = 0.027) with a QRS duration of 148±27 ms.

**Table 1:** Demographic and clinical characteristics of the study population.

Variable	Marfan	Controls	p-value
Age	24.82±15.44	20.13±7.81	0.262
Male	26	28	0.184
Height (cm)	173.99±22.18	168.55±14.50	0.020
Weight (kg)	18.50±29.15	60.95±16.33	0.224
BSA (m^2^)	1.70±0.45	1.64±0.27	0.224
Heart rate (bpm)	73.60±13.74	67.26±12.73	0.027
Systolic blood pressure (mm Hg)	115.12±19.06		
Diastolic blood pressure(mm Hg)	66.45±10.13		
Mitral valve prolapse	16		
Mild mitral regurgitation	14		
Mild aortic regurgitation	13		
Aortic valve ring diameter (mm)	22.61±4.10	21.28±3.19	0.180
Aortic root diameter (mm)	34.12±9.35	26.07±3.63	0.0001
ST junction diameter (mm)	26.73±7.26	20.55±3.60	0.002
ß blocker treatment	9		
ARB treatment	29		

*Abbreviations*: *BSA*: *body surface area*, *ST*: *sino-tubular*, *ARB*: *angiotensin receptor blocker*.

Forty-three patients were in NYHA functional class I and two were in functional class II. Nine (20%) patients were taking a beta-blocker, while an angiotensin receptor blocker (ARB) was part of the regular medication in 29 (64%) of the patients.

Genetic evaluation to identify the mutation of fibrillin-1 gene was done in 32 (71%) patients. In the 13 other patients FBN1 mutation analysis was not performed, due either to refusal of genetic testing by the patient or his/her parents or loss of contact after testing was recommended or the test results were unavailable.

The patients who performed genetic testing were biologically stratified into two groups: mutation types that typically lead to a missing or incomplete gene product were regarded as “severe”(n = 17, 53%). This included large (>1 exon) or out-of-frame deletions/insertions, nonsense point mutations and splice site mutations at <10 bp from exon predicted to cause exon skipping. Conversely, mutation types that typically lead to a complete yet structurally altered gene product were regarded as “mild”(n = 15, 47%). This included missense point mutations and in-frame deletions/insertions [22].

### 2. Evaluation of the left ventricular function using conventional echocardiography

The results of conventional echocardiography in patients and controls are summarized in Table 2.

**Table 2:** Left ventricular function among Marfan syndrome patients (MFS) and controls evaluated by conventional echocardiography.

Variable	MFS patients	Controls	p-value
M-mode LV EDD (mm)	9.79±2.56	9.19±1.58	0.291
M-mode LV ESD (mm)	31.98±8.14	30.33±4.13	0.511
LV FS (%)	37.33±7.05	37.31±4.35	0.686
MAPSE (mm)	17.31±3.31	19.00±2.49	0.005
LV E/A ratio	1.68±0.65	2.09±0.57	0.001
LV ICT (ms.)	46.18± 22.18	43.05±15.82	0.581
LV IVRT (ms.)	29.91±23.33	42.77±24.13	0.019
LV Tei index	0.25±0.11	0.28±0.09	0.247
Aortic VTI (cm)	25.37±11.91	27.06±4.66	0.001

*Abbreviations*: *LV*: *left ventricle*, *EDD*: *end-diastolic dimension*, *ESD*: *end-systolic dimension*, *FS*: *fractional shortening*, *MAPSE*: *mitral annular plane systolic excursion*, *E*: *early diastolic*, *A*: *late diastolic*, *ICT*: *isovolumetric contraction time*, *IVRT*: *isovolumetric relaxation time*, *VTI*: *velocity time integral*.

Fourteen patients (31%) had mitral valve prolapse with mild degree mitral valve regurgitation. The dimension of the aortic root in the MFS patients was 34.12±9.35 mm; 19 patients (42%) had a significantly dilated aortic root (defined as an aortic root dimension > mean±2SD of the aortic root among controls). None of the patients had significant aortic regurgitation.

Regarding the left ventricular systolic function using conventional methods, in comparison with the controls, no statistically significant difference was found in the patients regarding the M-mode derived LV FS and Doppler derived LV Tei index. On the other hand, the M-mode derived MAPSE (p = 0.005, Table 2) and Doppler derived aortic velocity time integral (p = 0.001, Table 2), were significantly lower in patients than in controls.

From the diastolic point of view, the Doppler derived E/A ratio was statistically significantly lower in patients than controls (p = 0.001, Table 2) and the LV isovolumetric relaxation time (IVRT) was also significantly reduced (p = 0.019, Table 2).

### 3. Evaluation of the left ventricular systolic function using three-dimensional derived ejection fraction and three dimensional speckle tracking

The 3D derived left ventricular volumes normalized to body surface area in patients and controls are given in Table 3. The inter-observer variability for LV end-diastolic volume, LV end-systolic volume and LV EF was 2±1 and 3±1 and 3±2%, respectively.

**Table 3:** Left ventricular function among Marfan syndrome patients (MFS) and controls as evaluated by 3-D echocardiography.

Variable	MFS patients	Controls	p-value
LV EDV/BSA (ml/m^2^)	77.34 ±24.19	64.95±7.73	0.012
LV ESV/BSA (ml/m^2^)	33.95±15.67	24.94±4.92	0.005
LV EF (%)	57.43±7.51	62.69±4.76	0.000
LV CO (l/min)	5.05±1.85	4.41±0.91	0.173
LV global longitudinal strain (%)	14.85±2.89	17.90±2.01	0.000
LV global circumferential strain (%)	13.93±2.81	16.82±2.17	0.000
LV global area strain (%)	25.76±4.43	30.51±2.61	0.000
LV global radial strain (%)	38.66±8.77	47.41±5.85	0.000

*Abbreviations*: *LV*: *left ventricle*, *EDV*: *end-diastolic volume*, *ESV*: *end-systolic volume*, *BSA*: *body surface area*, *EF*: *ejection fraction*, *CO*: *cardiac output*.

Patients had significantly lower 3D derived left ventricular ejection fraction (LVEF) than controls (p = 0.0001, Table 3).

No statistically significant difference was found between patients with and without an operation regarding LVEF (56.67±6.40 vs. 59.17±9.72%, p = 0.29).

The LVEF among patients correlated significantly with the diameter of the aortic root (r = -0.4, p = 0.013) but not with age or heart rate. MFS patients with an aortic root within the normal limits still had a significantly lower LVEF than controls (59.14±7.38 vs. 62.69±4.76%, p = 0.033).

The global 3D derived longitudinal, circumferential, area and radial deformation were significantly lower among patients than controls (Table 3). The inter-observer variability for global longitudinal, circumferential and area strain was 2±1, 2±1 and 3±2, respectively.

No statistically significant difference was found between patients with and without operation regarding the 3D derived global longitudinal (14.83 ± 3 vs. 14.92 ± 2.68%, p = 0.75), circumferential (13.38 ± 2.58 vs. 15.25 ± 3.02%, p = 0.084), area (25.28 ± 4.42 vs. 26.92 ± 4.44%, p = 0.40) and radial strain (37.75 ± 8.52 vs. 40.83 ± 9.33%, p = 0.44).

In patients, the diameter of the aortic root correlated significantly with the global longitudinal strain (r = -0.61, p = 0.0001), global circumferential strain (r = -0.46, p = 0.011) and global area strain (r = -0.56, p = 0.0001). Compared to controls, patients with an aortic root within the normal limits still had lower global longitudinal strain (15.95±2.77 vs. 17.90±2.01%, p = 0.013), global circumferential strain (14.82±2.56 vs. 16.82±2.17%, p = 0.003) and global area strain (27.41±3.66 vs. 30.51±2.61%, p = 0.001).

In regard to the severity of FBN1 mutation, no statistically significant difference was found between patients with mild fibrillin-1 mutation (n = 15) and those with severe mutation (n = 17) regarding their age (23±15 vs. 23±13 years, p = 0.94). Among patients with a severe mutation, 14 (82%) were without operation and three patients (18%) with. Of the patients with mild mutation, 9 (60%) had been operated upon and 6 (40%) had not. No significant difference was found between the severity of mutation and the need for surgery (p = 0.17).

**Fig 1:**
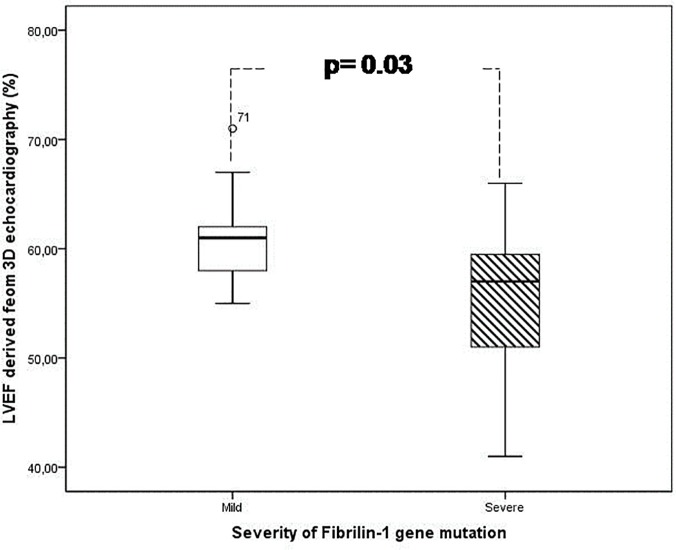
The reduced LV systolic function as assessed by three-dimensional echocardiography is related to the severity of gene mutation. LVEF: Left ventricular ejection fraction

**Fig 2:**
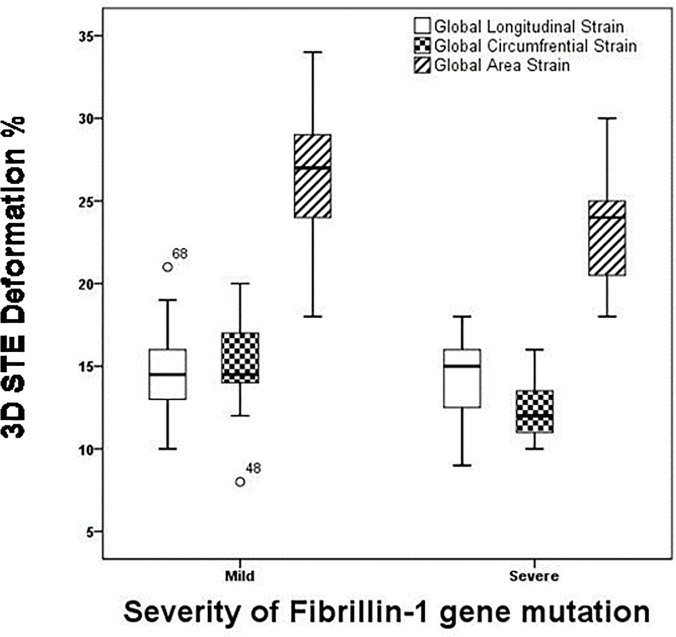
The reduced LV circumferential and not longitudinal systolic deformation as assessed by three-dimensional speckle tracking echocardiography is related to the severity of gene mutation. 3-D: Three-dimensional echocardiography, STE: Speckle tracking echocardiography.

MFS patients with a severe form of fibrillin-1 gene mutation had significantly reduced LVEF (60.92±4.71 vs. 55.20±7.03%, p = 0.033, Fig 1), reduced global circumferential strain (15.00±3.16 vs. 12.33±1.95%, p = 0.01) and global area strain (26.93±4.29 vs. 23.53±3.64%, p = 0.03, Fig 2) when compared to those with a mild form of fibrillin -1 gene mutation. On the other hand, the global longitudinal strain did not differ significantly between those with mild and severe mutation (14.85±2.93 vs. 14.07±2.55%, p = 0.71,Fig 2).

The multivariate linear regression analysis included the following four variables: patient age, mutation severity, surgical intervention and diameter of the aortic root. The severity of the gene mutation had a significant influence on the 3D-STE derived LVEF (p = 0.011), the circumferential strain (p = 0.024)) and mean AS (p = 0.03). On the other hand, the age, surgical interference and the diameter of the aortic root did not have a statistically significant influence on the 3D derived systolic parameters of LV function.

**Fig 3:**
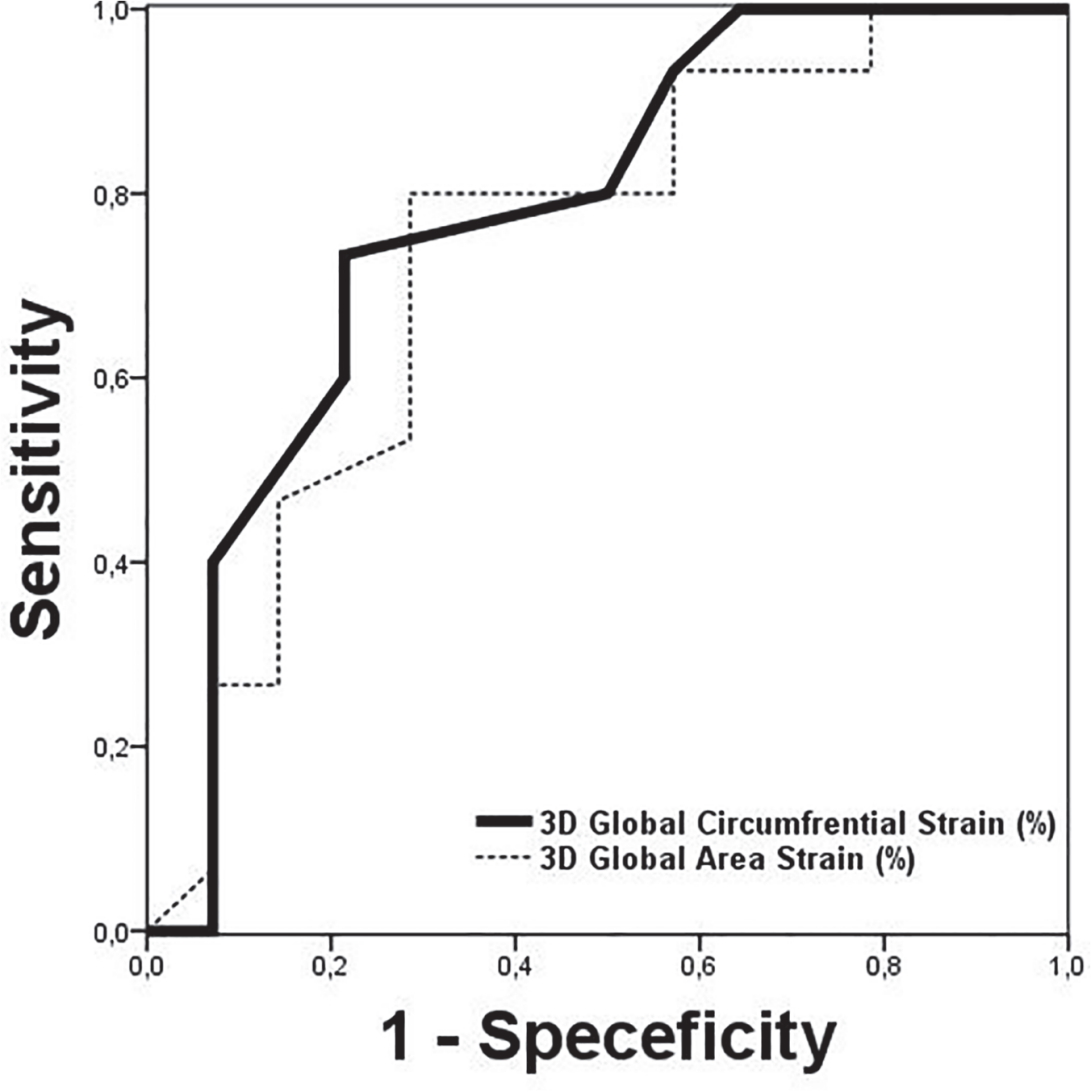
For prediction of severe gene mutation, the area under the ROC curve was 0.838 for global 3D derived LV circumferential strain and 0.790 for global 3D derived area strain.

For prediction of genetic type and the severity of gene mutation in MFS patients with genetic analysis (n = 32), the area under the ROC curve was 0.838 for global 3D derived LV circumferential strain and 0.790 for global 3D derived area strain. For identifying severe gene mutation; a global 3D derived LV circumferential strain of less than 13.5% had 73% sensitivity and 85% specificity, while global 3D LV area strain of less than 25.5% showed 80% sensitivity and 77% specificity (Fig 3).

### 4. Evaluation of the LA volumes and functions using three-dimensional derived echocardiography

The inter-observer variability for LA end-diastolic volume and end-systolic volume was 3 ± 2 and 2 ± 1% respectively. Compared to controls, patients had significantly larger LA end-diastolic volume (p = 0.001, Table 4), significantly reduced LA reservoir function (p = 0.003, Table 4) and statistically indifferent LA pump function (Table 4). The severity of the fibrillin-1 gene mutation was not related to the LA reservoir function or to the LA pump function. A significant negative correlation existed among the patients between the reservoir function of LA and their age (r = -0.55, p = 0.0001) and the diameter of the aortic root (r = -0.43, p = 0.009). Patients who had undergone surgery compared to those who had not had significantly lower LA reservoir function 39.59±13.49 vs. 47.32 ±12.65%, p = 0.003), LA EF (55.95±10.26 vs. 62.79±12.00% p = 0.0001) and statistically indifferent LA pump function (26.85±9.78 vs. 30.37±7.51%, p = 0.10). The LA pump function among patients was not related to age; on the other hand it was related to the systolic (r = 0.45, p = 0.010) and diastolic blood pressure (r = 0.56, p = 0.001).

**Table 4:** Left atrial function among Marfan syndrome patients (MFS) and controls evaluated by 3-D echocardiography.

Variable	MFS patients	Controls	p-value
LA EDV/BSA (ml/m^2^)	11.77 ±7.23	7.71±2.14	0.001
LA ESV/BSA (ml/m^2^)	25.84±10.78	22.0±6.34	0.323
LA kick volume (ml/m^2^)	28.46±21.15	18.55±7.55	0.040
LA reservoir function (%)	39.59 ±13.49	47.32±12.65	0.003
LA pump function (%)	26.85±9.78	30.37±7.51	0.102
LA EF (%)	55.95±10.26	62.79±12.00	0.000

*Abbreviations*: *LA*: *left atria*, *EDV*: *end-diastolic volume*, *ESV*: *end-systolic volume*, *BSA*: *body surface area*, *EF*: *ejection fraction*.

## Discussion

Our data showed that subtle global left ventricular dysfunction is detectable among asymptomatic MFS patients using 3D echocardiography. The observed LV dysfunction was related to the severity of genetic mutation, suggesting possible primary cardiomyopathy in MFS patients.

### 1. Left systolic ventricular function in MFS patients

Although conventional echocardiography has dramatically improved the early diagnosis of cardiovascular manifestations in patients with MFS, it is still debatable whether latent ventricular dysfunction in these patients can be detected using conventional echocardiography. Accordingly, the data regarding assessment of left ventricular systolic function using conventional echocardiography varies considerably in the literature [23–26]. One of the reasons for this debate is the frequently distorted ventricular shape. In such clinical settings, we need a technique that directly measures the LV ejection fraction rather than a technique that estimates it through certain geometrical assumptions. The best echocardiography technique that can be applied to fulfill these requirements is 3D echocardiography, since it has been validated against the "gold standard" MRI with good limits of agreement [18, 27]. In the present study, using the novel techniques derived from 3D echocardiography functional left ventricular systolic dysfunction could be detected among asymptomatic MFS patients. These functional changes were not apparent using conventional echocardiography. Thus the present study confirms the superiority of 3D echocardiography in detecting subtle LV dysfunction not apparent by conventional echocardiography [28–30].

The diameter of the aortic root may reflect the previously reported reduced general aortic vascular elasticity in MFS patients. This reduction in aortic vascular elasticity might influence ventricular function because of increased after-load, through ventricular arterial coupling [31]. From the present study it was obvious that altered ventricular arterial coupling was not the only culprit for altered ventricular function since the correlation is rather weak and in multivariate linear regression analysis its influence on the LVEF and LV circumferential strain derived from 3-D statistically disappears. Moreover, MFS patients with an aortic root diameter within the normal range still had reduced left ventricular function when compared to controls. Thus, our study supports that of De Witte et al. [9], who demonstrated that the reduced LV systolic function in MFS patients was not due to reduced aortic elasticity.

Interestingly, the present study using a multivariate analysis showed that the left ventricular ejection fraction and circumferential deformation as assessed by 3D echocardiography were related to the severity of gene mutation of the fibrillin-1gene. These results support the recently published experimental data on mice with MFS which suggested the presence of structural and functional myocardial involvement related to the severity of fibrillin-1 gene mutation [32]. Fibrillin-1 is present in the myocardium as an integral part of the normal myocardial extra cellular matrix (ECM), and it is particularly found at sites where myocardial contraction transmits power to the ECM [33, 34]. Cook et al. demonstrated recently in mice with MFS that reduced fibrillin-1 production by cardiomyocytes is sufficient to precipitate dilative cardiomyopathy in otherwise normal mice. In addition, they established a causal relationship between a structurally deficient ECM, mechanically impaired muscle tissue and abnormal mechanosignaling by cardiomyocytes [32]. Furthermore, deficient fibrillin-1 leads to altered expression of transforming growth factor-β (TGF-β) in the ECM of the myocardium, which eventually leads to myocardial structural changes [35]. TGF-β is also known to be involved in fibrosis in pressure-loaded heart failure [36] and to be over-expressed in the myocardium of patients with idiopathic hypertrophic cardiomyopathy [37].

In the present study the 3D derived LV longitudinal deformation, though statistically lower in MFS patients than in controls, was not related to the severity of gene mutation. Unlike the right ventricle, which is mainly composed of longitudinally oriented myocardial fibers, the left ventricle has mainly circumferentially oriented myocardial fibers [38]. Accordingly, it is not surprising to find that the severity of gene mutation in the fibrillin-1 gene mainly affects the circumferential rather than the longitudinal deformation of the left ventricle. Similar findings were found among survivors of acute myeloid leukemia treated with anthracycline [39]. Therefore we hypothesize that among patients with subtle ventricular dysfunction a re-arrangement of circumferential fibers into a more longitudinal orientation might happen. This results in partial correction of the longitudinal function at the expense of circumferential function.

### 2. LV diastolic function and left atrial function in MFS patients

The present study confirms the presence of diastolic function abnormalities among MFS patients reported in previous studies [7, 40]. In our study, the reduced Doppler derived E/A ratio might reflect the presence of relaxation abnormality, while the reduced IVRT might point to additional reduced LV compliance.

In MFS patients, data regarding atrial function is sparse. From our study we conclude that the atrial reservoir function is reduced while the atrial pump function does not statistically differ from that of age-matched controls. Since the main drive of atrial reservoir function is the ventricular systolic function [41], we may speculate that the reduced reservoir function among MFS patients is a reflection of the reduced LV systolic function. In patients with impaired relaxation the atrial pump function unusually increases to compensate for the impaired relaxation [42]. In the present study, although MFS patients had an abnormal relaxation reflected by the reduced Doppler derived E/A ratio, no significant increase in the LA pump function was observed among them. This can be explained by the observed diminished compliance among the MFS patients [43]. Reduced LV compliance will result in an increased LA after-load, which prevents enhancement of LA pump function among MFS patients with an abnormal relaxation. Invasive measurement of the LVEDP is needed to confirm our speculations.

### 3. Limitations

Information about invasive pressure measurements is lacking since our study was non-invasive. Invasive pressure measurements could have given more insight into the ventricular and atrial functional parameters and their implications.

Unfortunately, measurement of biochemical markers for myocardial dysfunction in serum such as TGF beta or NT Pro BNP was not a part of this study. Further studies should target the relation between biochemical markers and severity of gene mutation as well as the ventricular function.

Although MFS is considered a rare disorder, the relatively small sample in the present study is another limitation which may affect the comparison within the subgroups of severity of gene mutation. Further multi-center studies are needed to pursue the effect of severity of gene mutation and LV dysfunction in this cohort.

## Conclusion

In asymptomatic MFS patients latent LV dysfunction is detectable using 3D STE. The LV dysfunction is mainly related the severity of gene mutation, suggesting possible primary cardiomyopathy in MFS patients. Follow-up studies with regard to the prognostic value of these observations are warranted.

## Supporting Information

S1 Dataset (ENL)
